# Death of Dr. Cooke

**Published:** 1854-01

**Authors:** 


### DEATH OF DR. COOKE.

DR. JOHN ESTEN COOKE, for many years connected with the Lexington Medical School, and subsequently with the Medical Institute of Louisville, died at his residence in Trimble county, on the 20th of October, in the seventieth year of his age. Dr. Cooke had long been in declining health, and believed himself to be the subject of pulmonary consumption. The state of his health had rendered him incapable of pursuing his profession for several years before he died, and he had retired to a farm on the banks of the Ohio river.

Dr. Cooke's father having been a physician, it is probable that his mind was turned at a very early period to the profession of medicine, in which he was carefully and thoroughly educated. He took his degree as doctor of medicine, at the University of Pennsylvania, in 1805. Dr. Cooke had acquired a high reputation as a practitioner and writer before he was invited, in 1827, to the Chair of Theory and Practice in Transylvania University. A paper which he wrote on "Autumnal Bilious Fever," published in the American Medical Recorder, in 1822, evinced reading, observation, and uncommon powers of analysis, as well as a boldness of practice which was greatly esteemed at that day. At the time he was elected to the Professorship in the School at Lexington he had made considerable progress in the organization of a Medical School at Winchester, Virginia. He came to Lexington as the successor of DR. DRAKE. His first course of Lectures was one of the most remarkable ever listened to by a class of Medical Students. Discarding all systems of Nosology, Dr. Cooke presented a classification of his own. He had a theory which he believed explained the phenomena of nearly every disease, and his lectures were taken up with an exposition of his theory and the practice founded upon it. The theory was a very simple one, and his practice as simple:—that disease depends upon venous congestion, and is to be cured by remedies which act upon the liver. This is a statement of his views of the pathology, not only of fevers, cholera, dysentery, &c., but of menorrhagia, dysmenorrhœa, and most of the complaints of females. A view of pathology so simple could not fail to be attractive to his pupils, and it is safe to say that few teachers have exerted a greater sway over medical theory and practice than was exercised by Dr. Cooke for many years in the valley of the Mississippi. His mind was eminently a logical one, and as honest in its convictions as forcible in its logic. His array of facts was strong, and his hearers had little disposition to resist the force of an argument which conducted them to so satisfactory a theory, and a practice which, in the hands of their teacher, had succeeded so well. It was Dr. Cooke's happy mental constitution to be thoroughly convinced of the truth of whatever he adopted. Whether on matters of theology or of medicine he had no half-formed opinions; with him it was entire belief or utter rejection. Few men have ever entertained so undoubting a faith in the certainty of medicine; and this was probably a main element of his success in carrying conviction so generally to the minds of his students. They saw at once that he was perfectly honest, and this predisposed them strongly in favor of his views. They were averse to rejecting doctrines which so ingenuous a man held so firmly and enforced by arguments so fair and convincing.

In his intercourse with men, as in his public instruction, this simplicity and candor was Dr. Cooke's ruling trait of character. Honesty, perfect ingenuousness, unswerving integrity, this it was that marked his conduct on all occasions and singled him out among men. He was not fluent as a lecturer; he made not the slightest pretensions to eloquence, and in his manners he was blunt and sometimes abrupt, but the kindness of heart which was visible beneath this unpromising exterior, and the air of sincerity which pervaded all that he said or did, caused those around him to forget the manner, and compelled belief in his statements. He had true warmth of character, and in his attachments he was decided, ardent, and steady. We have alluded to the strength and clearness of his convictions. He was a true Christian. Not a doubt appeared ever to disturb the deep serenity of his religious faith.

Dr. Cooke was a voluminous writer. Besides his treatise on Pathology and Therapeutics in two large volumes, he wrote copiously for the Transylvania Journal of Medicine, of which he was one of the editors, and also employed his pen occasionally upon theological subjects. His writings are a model of clearness, directness, and simplicity of style, and those on medicine abound in valuable facts and original observations.

When the writer of this brief notice read to the medical class in the University of Louisville, a little more than a year ago, a history of the medical department of that Institution, all the men who had held professorships in it were still alive. Three of the number have since passed away. Of these DR. DRAKE and DR. COOKE were of nearly the same age. They were students in the same class in Philadelphia, in 1805. DR. CALDWELL was at that time, a teacher of note, and a writer widely known. There is something impressive in the demise of these eminent men, who had sustained to each other, at different periods of their lives, the relations of colleague, and of pupil and preceptor, in such quick succession. They have left behind them few American physicians who have occupied so large a space in the professional mind of our country.

